# 342. Secondary Infections following Tocilizumab for Treatment of COVID-19 Pneumonia

**DOI:** 10.1093/ofid/ofab466.543

**Published:** 2021-12-04

**Authors:** Adam Warner, Mujahed Abbas, Benjamin S Avner, Thomas E Flynn, Todd A Walroth, Brett W Jagger

**Affiliations:** 1 Bronson Healthcare Group, Kalamazoo, Michigan; 2 Western Michigan University - Homer Styrker School of Medicine, Kalamazoo, Michigan; 3 Eskenazi Health, Indianapolis, Indiana; 4 Western Michigan University Homer Stryker MD School of Medicine, Kalamazoo, Michigan

## Abstract

**Background:**

Guidelines recommend use of tocilizumab (TCZ), an inhibitor of pro-inflammatory IL-6 signaling, for hospitalized patients with progressive severe or critical Coronavirus disease 2019 (COVID-19). The incidence of infectious complications following the use of TCZ for COVID-19 has not been well-described.

**Methods:**

We conducted a retrospective, observational matched cohort study of severely ill, hospitalized adult patients with confirmed COVID-19 who were treated with TCZ between 2/24/2021 and 6/1/2021. The intervention group, comprised of patients who received a single dose of TCZ, was matched based on c-reactive protein (CRP) and fraction of inspired oxygen (FiO_2_) with a control group who did not receive TCZ, and were hospitalized between 10/12/2020 and 3/6/2021. The primary outcome of the study was diagnosis of a bacterial or fungal infection after day 3 of the index hospitalization. Secondary outcomes included all-cause mortality during the study period and length of stay.

**Results:**

75 patients who received TCZ were identified during the study period, and matched 1:1 with 75 control patients. Baseline CRP and FiO_2_ were similar between groups, while the median age in the TCZ group was younger (61 versus 71 years), reflecting the epidemiology of the outbreak during the TCZ and control study periods. 15 bacterial and fungal infections were identified in the TCZ group (20.0%) and 4 (5.3%) infections in the control group (p = 0.012). The majority of infections in both groups were bacterial, with a disproportionate number of bloodstream infections in the TCZ group [7 (9.3%) vs 2(2.6%); p = 0.166]. 28 patients (37.3%) died in the TCZ group compared to 39 (52.0%) in the matched control (p = 0.068). Median time to discharge was similar between TCZ and control patients (11 versus 12 days; 95% CI -2,2).

**Conclusion:**

Secondary infections in adult patients with severe and critical COVID-19 were more common in patients who had received TCZ. Prospective studies are needed to confirm and further describe this association.

**Disclosures:**

**All Authors**: No reported disclosures

